# Coronaviral RNA-methyltransferases: function, structure and inhibition

**DOI:** 10.1093/nar/gkab1279

**Published:** 2022-01-08

**Authors:** Radim Nencka, Jan Silhan, Martin Klima, Tomas Otava, Hugo Kocek, Petra Krafcikova, Evzen Boura

**Affiliations:** Institute of Organic Chemistry and Biochemistry of the Czech Academy of Sciences, Flemingovo nam. 2, 166 10 Prague 6, Czech Republic; Institute of Organic Chemistry and Biochemistry of the Czech Academy of Sciences, Flemingovo nam. 2, 166 10 Prague 6, Czech Republic; Institute of Organic Chemistry and Biochemistry of the Czech Academy of Sciences, Flemingovo nam. 2, 166 10 Prague 6, Czech Republic; Institute of Organic Chemistry and Biochemistry of the Czech Academy of Sciences, Flemingovo nam. 2, 166 10 Prague 6, Czech Republic; Institute of Organic Chemistry and Biochemistry of the Czech Academy of Sciences, Flemingovo nam. 2, 166 10 Prague 6, Czech Republic; Institute of Organic Chemistry and Biochemistry of the Czech Academy of Sciences, Flemingovo nam. 2, 166 10 Prague 6, Czech Republic; Institute of Organic Chemistry and Biochemistry of the Czech Academy of Sciences, Flemingovo nam. 2, 166 10 Prague 6, Czech Republic

## Abstract

Coronaviral methyltransferases (MTases), nsp10/16 and nsp14, catalyze the last two steps of viral RNA-cap creation that takes place in cytoplasm. This cap is essential for the stability of viral RNA and, most importantly, for the evasion of innate immune system. Non-capped RNA is recognized by innate immunity which leads to its degradation and the activation of antiviral immunity. As a result, both coronaviral MTases are in the center of scientific scrutiny. Recently, X-ray and cryo-EM structures of both enzymes were solved even in complex with other parts of the viral replication complex. High-throughput screening as well as structure-guided inhibitor design have led to the discovery of their potent inhibitors. Here, we critically summarize the tremendous advancement of the coronaviral MTase field since the beginning of COVID pandemic.

## INTRODUCTION

Coronaviruses (CoVs) are important human and animal pathogens that belong to positive-sense single-stranded RNA (+RNA) viruses. CoVs refer to members of the subfamily *Coronavirinae* of the family *Coronaviridae*, order *Nidovirales*, which belong to the realm *Riboviria*. We can distinguish four genera of CoVs which are named according to the Greek alphabet: Alpha-CoV, Beta-CoV, Gamma-CoV and Delta-CoV. Whereas Alpha-CoVs and Beta-CoVs contain a large number of mammal and human pathogens, their Gamma and Delta siblings are mostly avian, although some of them can be found in cetaceans and pigs, respectively (1).

Currently, only seven coronaviruses are known to infect humans, causing a wide range of disease severity. Human coronaviruses (HCoVs) HCoV-229E and HCoV-NL63 belong to Alpha-CoVs, and they are mostly responsible for milder forms of upper respiratory illnesses. However, both of these viruses have been implicated in the pathogenesis of rather severe diseases such as pneumonia, usually in immunocompromised patients (2,3). Also, HCoV-OC43 and HCoV-HKU1 that belong to the Beta-CoVs are widespread human viruses that are associated with common cold-like symptoms. However, the rest of the Beta-CoVs that have been shown to attack humans are very important threats to human health. In 2002, an outbreak of illness connected to a life-threatening pneumonia emerged in the Guangdong province of China. This disease was associated with a novel Beta-CoV that was later named severe acute respiratory syndrome coronavirus, or SARS-CoV. In 2002 and 2003, isolated cases of this disease were reported in more than 30 countries. Luckily the spread was stopped before it could cause a global pandemic. Ten years later, another Beta-CoV caused an outbreak of Middle East respiratory syndrome (MERS) that affected people in 26 countries and had an even higher fatality rate of more than 30%. Most recently, in December 2019, numerous cases of severe lower respiratory illness were reported by officials in China. These patients were shown to be infected by yet another Beta-CoV that was closely related to SARS-CoV. Therefore, this virus was named SARS-CoV-2. Unlike SARS-CoV and MERS-CoV, the spread of this virus has not been contained, and the virus has spread worldwide, causing a global pandemic. As of November 2021, 260 million cases of COVID-19 have been confirmed, which has resulted in over 5.1 million deaths around the globe (covid19.who.int). These facts clearly indicate that we were woefully unprepared to combat these insidious pathogens, and we must take steps in the future to respond more quickly to combat coronavirus pandemics. Clearly, this can only be achieved through a thorough understanding of the pathological processes that these viruses cause, on the basis of which we will be able to design effective therapeutic or preventive approaches to inhibit the spread of these viruses in the population.

The key to understanding these viruses is, of course, understanding their genetic information and elucidating the role of the individual proteins that these viruses encode for their pathogenesis. In this review, we focused on advances in the study of coronaviral methyltransferases (MTases) that are essential for the installation of the viral RNA cap, with a special focus on their potential as molecular targets for future therapeutic intervention. Therefore, a significant portion of this review article is devoted to structural studies of these enzymes and to already known inhibitors that may serve as starting points for the preparation and optimization of therapeutic compounds.

### RNA capping pathway

Eukaryotic mRNA possesses a special chemical structure, called a cap, on its 5′ end. This RNA cap is important for at least four reasons: (i) mRNA-splicing, (ii) mRNA-stability, protecting its 5′ end from premature degradation by exonucleases, (iii) mRNA transport from nucleus and (iv) efficient mRNA translation (4,5).

**Figure 1.**
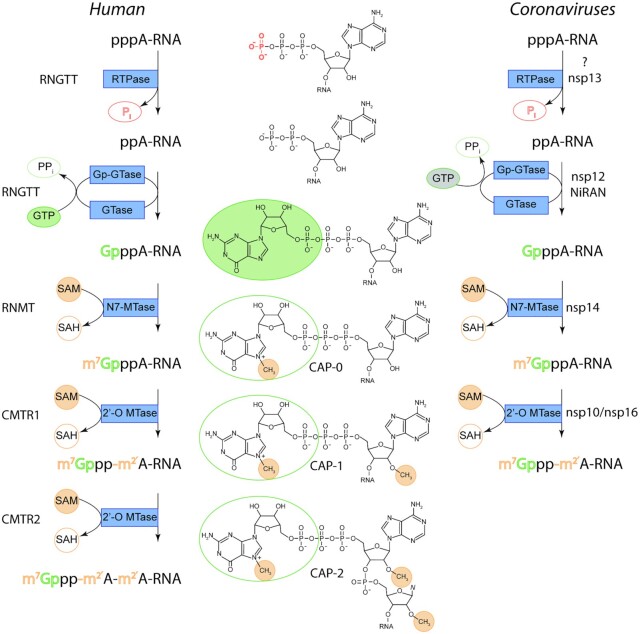
Comparison of human and coronaviral mRNA cap formation. RNGTT - RNA guanylyltransferase and 5′-phosphatase, RNMT, RNA guanine-7 methyltransferase; CMTR1, Cap methyltransferase 1; CMTR2, Cap methyltransferase 2.

Several enzymatic activities are needed to attach the cap (Figure 1). The first two steps are catalyzed by the human capping enzyme that interacts with phosphorylated RNA-polymerase II ensuring the specificity of the process for mRNAs. The HCE N-terminal domain removes the γ-phosphate from the nascent pre-mRNA, and then its C-terminal domain catalyzes the transfer of GTP, yielding a Gppp-pre-mRNA. In the third step, the N7 position of the attached guanosine is methylated by the mRNA cap guanine-N7-MTase (RNMT), yielding an m^7^Gppp-pre-mRNA. This structure is referred to as cap-0 and it is the final product in lower eukaryotes such as the budding yeast. However, in humans, the cap-0 is further methylated by the cap-specific mRNA MTase 1 (CMTR1) at the 2′-*O*-ribose position of the first nucleotide giving rise to the cap-1 which can be further methylated by CMTR2 at the 2′-*O*-ribose position of the second nucleotide yielding a cap-2 (6).

Coronaviruses mimic this process to ensure stability of their RNA within the cell and their genomic and subgenomic RNA is capped (7). The presence of the cap in was first demonstrated in ^32^P-labeled murine hepatitis virus (MHV) RNA (8) and later confirmed by immunoprecipitation experiments in the related equine torovirus (9). The most significant difference is that coronaviruses synthesize their RNA cap in the cytoplasm where (not surprisingly) the RNA capping machinery co-localizes with dsRNA (10). Until recently, the enzyme catalyzing the first step(s) was not known (7), however the 5′-triphosphatase activity of the non-structural protein 13 (nsp13, a helicase) has been implicated (11). Yan *et al.* suggested that the first two steps are catalyzed by the helicase (γ-phosphate removal) and by the nsp12 NiRAN (Nidovirus RdRp associated nucleotidyl transferase) domain (GTP transfer) (12). Subsequently, mRNA is methylated on N7 of this guanine by the coronaviral N7-MTase (nsp14) which gives rise to a cap-0 structure. Subsequently, the coronaviral 2′-*O*-MTase (nsp10/nsp16 complex) carries out the next methylation of the 2′ OH ribose group of the first nucleotide resulting in cap-1 (13–15). This process might not be strictly directional as the coronaviral 2′-*O*-MTase also accepts the pre-cap structure as the substrate, albeit with much lower efficiency (16). Current structural models of the coronaviral replication elongation complex differ (12,17,18). However, all published structural models (12,17,18) suggest that the coronaviral RdRp and MTases form molecular complexes and that the newly replicated RNA (genomic and subgenomic) is capped and methylated while being synthesized.

Interestingly, other non-canonical cap structures have been recently described. For instance, NAD+, FAD, ADP-ribose and other molecules can be attached to the 5′ end of the bacterial or eukaryotic mRNA often by an *ab initio* mechanism when a DNA-dependent RNA-polymerase uses them as a substrate (19,20). These non-canonical caps have also been observed in viral genomic RNA. However, thus far their biological role and their role in viral replication has not been clearly defined (21).

### The RNA cap in the viral lifecycle

The RNA cap also plays a vital role in innate immunity. Several mechanisms that recognize non-capped ppp-RNA exist, and its recognition leads to induction of interferon genes and to the establishment of an antiviral state in the cells (22). Non-capped ppp-RNAs are one of the pathogen-associated molecular patterns (PAMP) that are recognized by pattern recognition receptors (PRRs) in cytoplasm.

**Figure 2.**
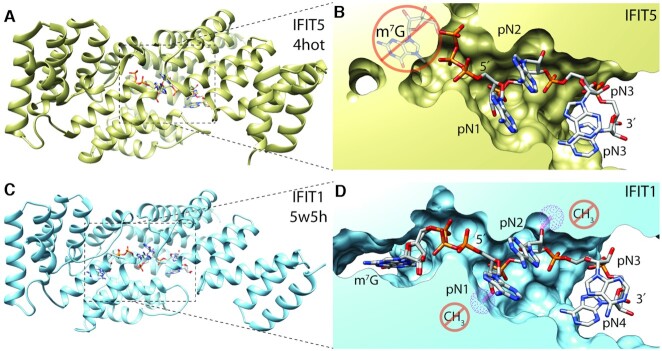
Recognition of ‘pre-cap’ and cap-0 structures by IFIT1 and IFIT5. (**A**, **C**) Comparison of homologous overall structures of IFIT5 (gold) and IFIT1(light blue). Both of these proteins have a deep RNA binding pocket (**B**, **D**). The pocket of IFIT1 cannot accommodate an RNA molecule bearing a cap-1 or cap-2 that have methylated 2′-OH ribose groups (clash space depicted as cyan spheres), while IFIT5 can only accommodate 5′-ppp-RNA.

Most importantly, in the case of uncapped RNA those PRRs are the RIG-I (retinoic acid-inducible gene I) and IFIT (interferon-induced protein with tetratricopeptide repeats) (23–25). mRNAs bearing just the cap-0 are recognized by a PRR called Mda5 (melanoma differentiation-associated protein 5), a member of the RIG-I-like receptor family (26). The mechanism of action of these PRRs differs. For instance, RIG-I is a helicase that recognizes 5′ - ppp-dsRNA (an intermediate of ssRNA virus replication) while IFIT5 has a deep RNA binding pocket. However, that pocket only accommodate a ppp-RNA molecule, not a molecule bearing any cap or even the pre-cap structure (Figure 2A, B). The sophisticated IFIT1 RNA binding site is able to distinguish between RNAs with cap-0 or cap-1/cap-2 (Figure 2C, D).

Nevertheless, activation of any of these anti-viral PRRs that recognize non-capped RNA leads to expression of interferon-stimulated genes (ISGs) and the induction of an anti-viral state. It is perhaps not surprising that viruses have evolved various intriguing mechanisms to protect and/or hide their RNAs (27). Installing a cap indistinguishable from the human cap is one of these mechanisms (28). The influenza virus, an orthomyxovirus, evolved an interesting mechanism called cap-snatching where it ‘steals’ the cap from host mRNAs (29). Many viral families including flaviviruses, coronaviruses, rhabdoviruses, paramyxoviruses, poxviruses and reoviruses developed their own capping machinery (28,30). This review is focused on the two most important players of the coronaviral capping machinery: the N7-MTase nsp14 (non-structural protein 14) and the 2′-*O*-MTase nsp16 (non-structural protein 16) (14,15,31–35). A special emphasis will be placed on the structures of these proteins and on structure-guided inhibitor design.

### Structure of the 2′-*O*-MTase: the nsp10/nsp16 protein complex

The coronaviral 2′-*O*-MTase is the nsp16 protein. However, it would be more accurate to describe the 2′-*O*-MTase as a heterodimeric protein complex composed of the catalytic subunit nsp16 and the activating subunit nsp10 because the nsp16 is inactive unless bound to this small activating protein (15). In this review, coronaviral 2′-*O*-MTase will always mean the nsp10/nsp16 protein complex.

**Figure 3.**
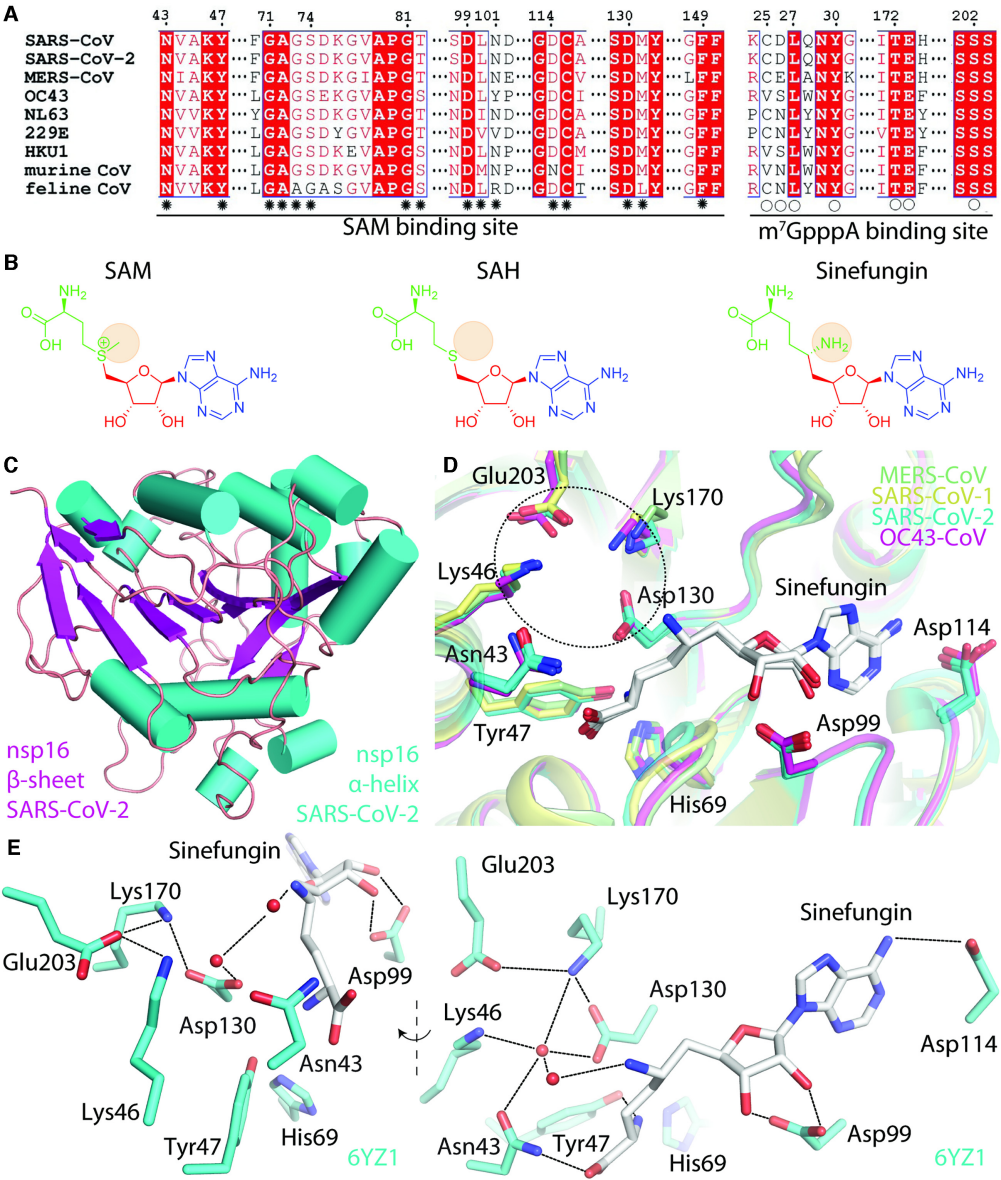
Coronaviral 2′-*O*-MTase - the nsp16 subunit. (**A**) Primary sequence alignment highlighting the most important residues. Residues forming the SAM and m^7^GpppA binding sites are marked by asterisks and circles, respectively. See Supplementary Figure S1 for full sequence alignment. (**B**) Structures of SAM, SAH, and sinefungin. Amino acid moiety is shown in green, sugar is in red, and the base is in blue. (**C**) Cartoon representation of nsp16 from SARS-Cov-2, the structure resembles a sandwich with a β-sheet in the middle (magenta) and the slices of bread made of α-helices (cyan). (**D**) Superposition of the known structures of coronaviral nsp16s revealed a very high conservation of the SAM binding and of the active site of this MTase. The catalytic tetrad (Lys46, Asp130, Lys170 and Glu203) is highlighted by a circle. (PDB IDs: MERS-CoV:5YNB, SARS-CoV:2XYR, SARS-CoV-2:6YZ1, OC43-CoV:7NH7). (**E**) Interactions of sinefungin with the active site of the enzyme (SARS-CoV-2). The amino acids involved in the interaction with sinefungin are shown as sticks, the water molecules are shown as red spheres, and selected hydrogen bonds are depicted as dashed lines. The left side of the panel shows to view along sinefungin, whilst the right side is rotated by ∼90° and down by ∼30°.

The structure of coronaviral 2′-*O*-MTase has been extensively studied in the last decade especially from the recently emerged and dangerous coronaviruses SARS, MERS, and SARS-2 (36–40) and recently also the structure of the OC43 2′-*O*-MTase has become available (41). All reported structures of coronaviral nsp16 proteins are in good agreement and they reveal a Rossmann or more specifically a Rossmann-like fold (36–43), which is very common for nucleotide-binding enzymes (44). This is the case with the coronaviral nsp16 as well, as it is composed of 12 α-helices and 12 β-strands (Figure 3, Supplementary Figure S1). The β-strands form an extensive central β-sheet in the shape of letter ‘J’ that is surrounded by α-helices on both sides (Figure 3B) creating a structure that resembles a sandwich with the β-sheet in the middle and the slices of bread made of α-helices.

The structural analysis also revealed a very high conservation of the SAM (*S*-adenosyl-l-methionine) binding site (Figure 3). In fact, all the residues that make direct contact or have a water bridge with SAM are conserved. This conservation has important implications for drug development because it suggests that a compound targeting the SAM binding site would be active against most, if not all, coronaviruses.

### Mechanism of the 2′-*O*-methylation reaction

**Figure 4.**
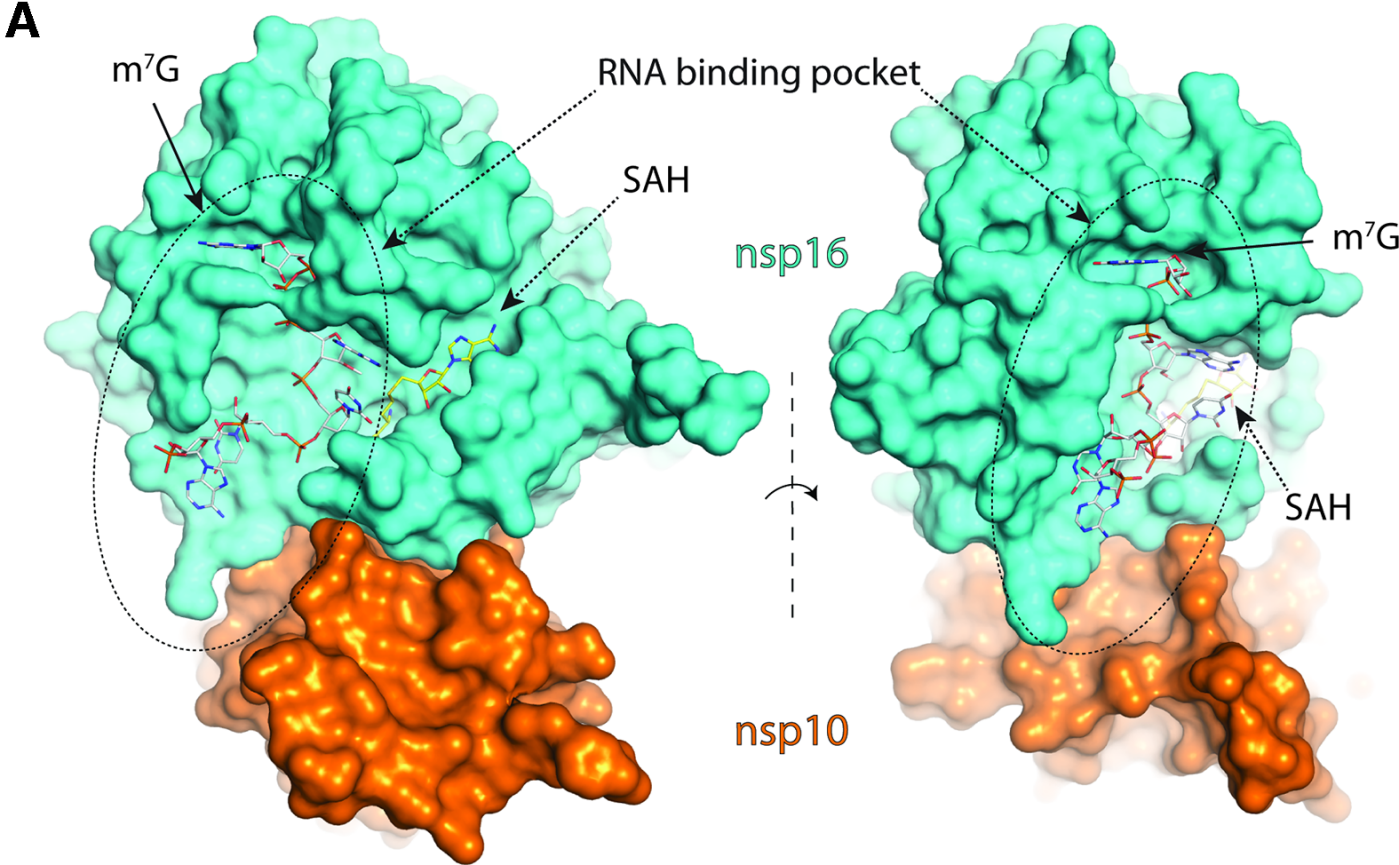
RNA binding towards the coronaviral 2′-*O*-MTase. The crystal structure of nsp10/nsp16 from coronavirus SARS-CoV-2 with cap-1 as the product along with SAH (PDB ID: 7L6R). Nsp16 is in cyan and nsp10 in orange, m^7^G is locked in the tunnel of a long RNA binding pocket spanning across the nsp10/nsp16 heterodimer.

Deep insights into the mechanism of methyl transfer by the 2′-*O*-MTase were obtained by recent crystal structures of the SARS-CoV-2 enzyme. First, it was shown that the putative RNA binding site is a large canyon, localized mostly on the nsp16 subunit (Figure 4). Modeling revealed that approximately five nucleotides can occupy the RNA binding channel, positioning the ribose 2′ hydroxyl group within close proximity to the SAM methyl group (38,40,42).

**Figure 5.**
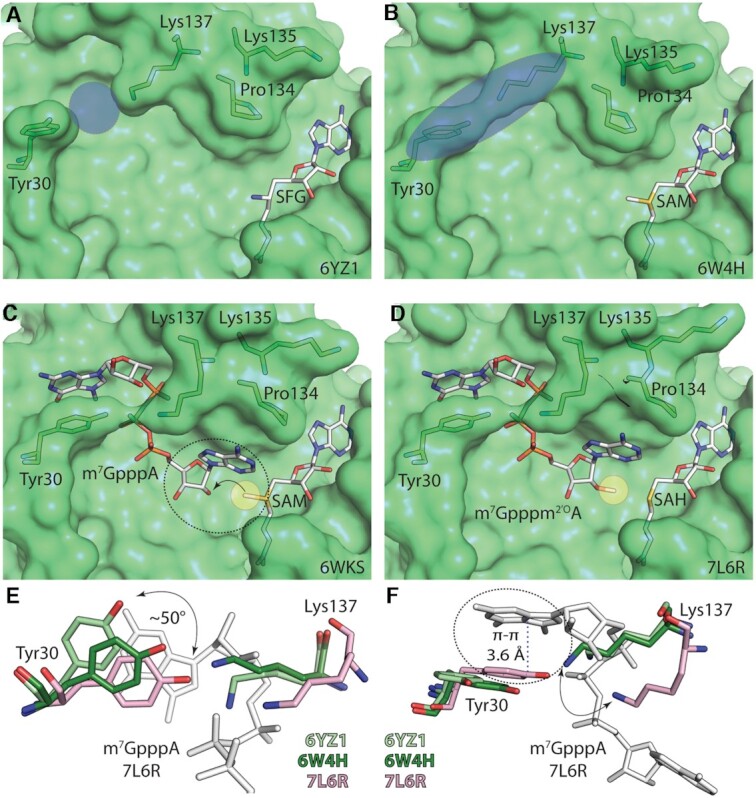
Crystallographic snapshots of methylation and conformational changes during cap-1 synthesis by coronaviral 2′-*O*-MTase. (**A**, **B**) Opened and closed conformation of Tyr30 and Lys137 of nsp16. (**C**) Cap-1 locked in the RNA binding pocket by the action of Tyr30 and Lys137 whilst Pro134 and Lys135 are reorganized to accommodate the first nucleoside of RNA. The methyl group of SAM (yellow sphere) is ready to be transferred on the 2′*-O* position of the ribose ring. (**D**) Methylated ribose forms a complete cap-1 RNA. (**E**, **F**) Detailed view of the interaction and the degree of movements between residues Tyr30 and Lys137 in nsp16. Paired residues from individual crystal structures are color matched. Both RNA bound structure (PDB IDs: 6WKS and 7L6R) display identical orientation of these two residues whilst locking m7G. RNA is in white sticks for clarity.

Further insights were obtained from a series of crystal structures of this 2′-*O*-MTase in complex with the methyl donor SAM and the accepting cap-0 analog captured by serial crystallography at room temperature (42). This study was able to capture three states (i) cap-0 bound, (ii) cap-0 and SAM bound and (iii) cap-1 and SAH (*S*-adenosyl-l-homocysteine) bound. This study provided a nice confirmation of previous studies based on a single frozen crystal. The structures obtained at room temperature were almost identical, and revealed the movement of the two gate loops (AAs 28–35 and 131–146). This conformational change leads to the formation of a hydrogen bond between Tyr30 and Lys137 which is necessary to create the cap-0 binding site (Figure 5). However, the SAM binding site exhibits no conformational changes between SAM, SAH or sinefungin bound structures (38,42) making it difficult to explain how the SAH/SAM exchange is controlled. It was suggested that it could be controlled by nsp10 dissociation/re-association with the nsp16 subunit (42). In this model, only the nsp10/nsp16 complex would bind the SAM/SAH and when the complex dissociates and so does the small molecule co-factor. This model is supported by the observation that SARS-CoV-2 nsp16 does not bind SAM unless in complex with nsp10 (36). *In silico* analysis based on over 1 ms molecular dynamics suggests that Nsp10 shifts Nsp16’s conformational ensemble to stabilize more open SAM- and RNA-binding pockets (45).

**Figure 6.**
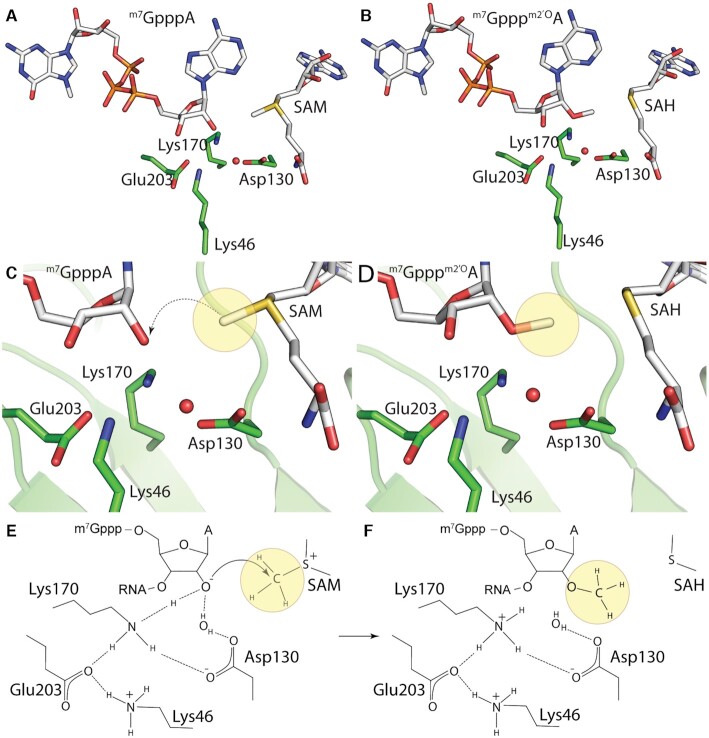
Mechanism of the 2′-*O* methylation reaction. (**A**) view on the active site with SAM, the catalytic tetrad (Lys46, Asp130, Lys170 and Glu203) and the substrate RNA molecule (cap-0), (**B**) identical view on the product cap-1, active site and the side product SAH. (**C**, **D**) detailed view on catalytic site and the ribose where a methyl group (yellow sphere) is transferred. (**E**, **F**) Model of the catalytic mechanism based on the crystal structures (PDB IDs:6WKS and 7L6R).

The actual catalytic reaction is catalyzed by the catalytic tetrad Lys-Asp-Lys-Glu (Lys46-Asp130-Lys170-Glu203 in SARS-CoV-2) and requires a divalent ion, Mg^2+^ or Mn^2+^. However, it appears that this cation is not directly necessary for the catalytic process itself, but is involved in the correct positioning of the methylated RNA (43). The actual mechanism of the reaction, which is chemically an S_N_2 reaction, is then apparently mediated by Lys170, which acts as a necessary base, in an ongoing nucleophilic substitution in which SAM plays the role of methyl group donor (Figure 6).

**Figure 7.**
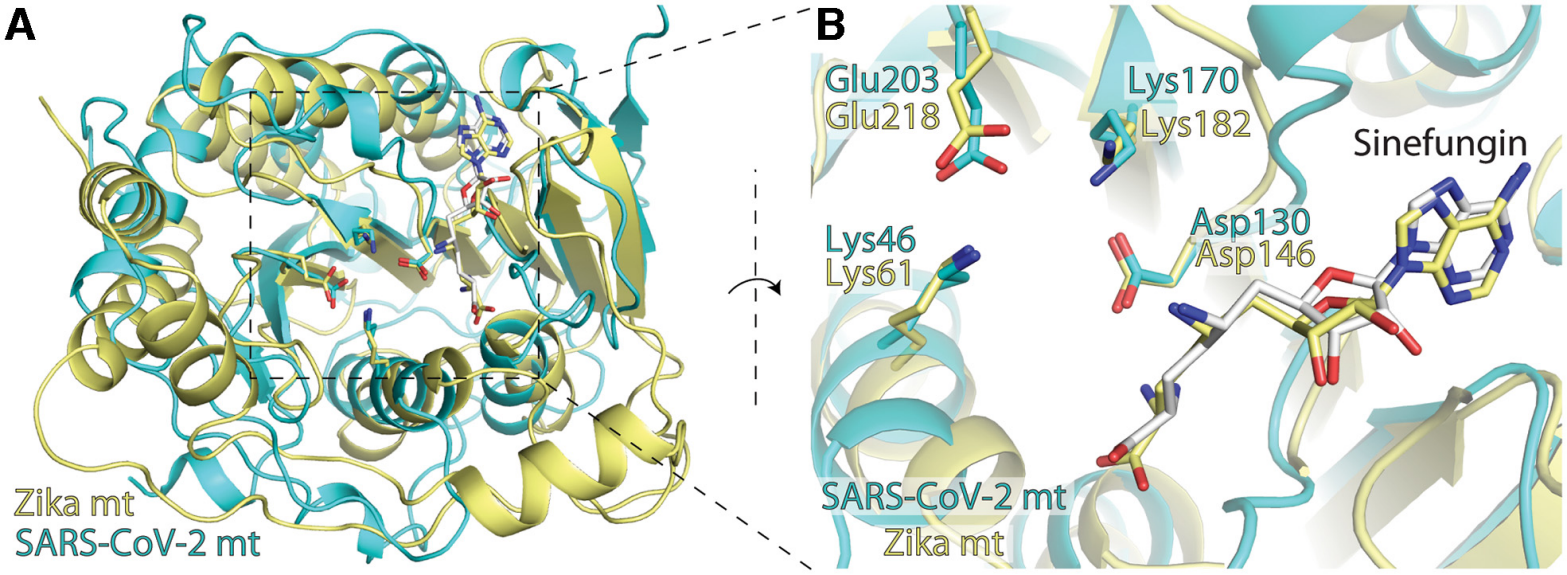
Structural alignment of MTases from ZIKV and SARS-CoV-2. (**A**) overall structures of ZIKV (yellow) and SARS-CoV-2 (cyan) MTases (**B**) conserved residues of the catalytic tetrad from both enzymes in the vicinity of sinefungin.

While the fold of the 2′-*O*-MTase is well conserved among coronaviruses, the similarity of the overall fold with other 2′-*O*-MTases from +RNA viruses from different orders such as the Zika virus (family *Flaviviridae*, order Amarillovirales) is rather small (Figure 7A). However, the position of the catalytic tetrad, Lys61-Asp146-Lys182-Glu218 in the case of Zika virus (46), is perfectly conserved (Figure 7B) illustrating the same mechanism of the methyl transfer reaction between these two viruses. This also suggests that development of antivirals targeting the 2′-*O*-MTase from different families and/or orders of +RNA viruses should be in principle possible. However, close attention must be paid to selectivity because this catalytic tetrad is also conserved in the human 2′-*O*-MTase CMTR1 but not in other human MTases (Supplementary Figure S3).

### Structure of nsp14

**Figure 8.**
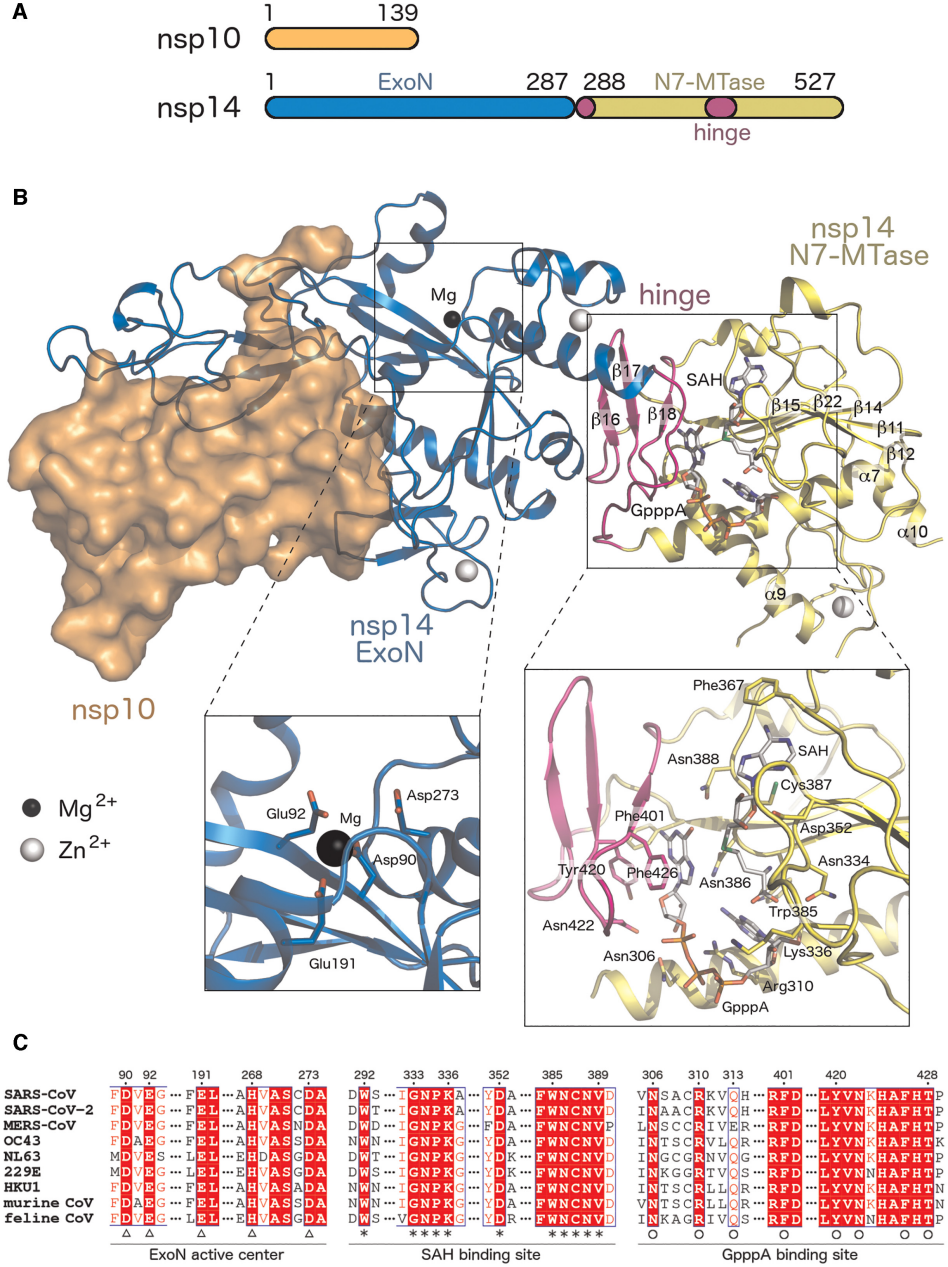
Structure of the coronaviral nsp10/nsp14 complex. (**A**) Schematic representation of the domain structure of the SARS-CoV nsp14/nsp10 protein complex. (**B**) The overall fold of the SARS-CoV nsp14/nsp10 protein complex and detailed views of the ExoN active site and the SAH- and GpppA-binding sites (based on PDB ID: 5C8S). (**C**) Sequence alignment of the active sites of selected coronaviral nsp14 proteins. Residues forming the ExoN domain active center and the SAH- and GpppA- binding sites are marked by triangles, asterisks, and circles, respectively. See SI Fig. 2 for full sequence alignment.

Nsp14 a bifunctional protein bearing two enzymatic activities: N7-MTase and 3′ → 5′ exonuclease (ExoN) activity. Each activity is associated with one of its two domains: the N-terminal domain bears the ExoN and the C-terminal domain bears the MTase activity. These two domains are connected with a small hinge region which is actually located within the MTase domain (the flexible loop Lys288–Gly300 and three small β-sheets Leu406–Ala430) (Figure 8) (47). Small-angle X-ray scattering (SAXS) is the method of choice to characterize flexible proteins and protein complexes too large for NMR analysis (48). Ferron *et al.* used this method, and their SAXS analysis revealed flexibility of nsp14 (47). This could be the reason the full-length SARS-CoV-2 nsp14 has resisted all efforts to obtain a crystal structure to date, however, the ExoN domain can be crystallized (49). Fortunately, cryo-EM structures featuring full length nsp14 have recently become available (17,50).

Both enzymatic activities of nsp14 are being considered as targets for antivirals. In addition, due to high conservation of nsp14 between individual coronaviral species (Supplementary Figure S2), nsp14 is a promising target for the design of inhibitors with the potential to act as pan-coronavirus drugs.

#### The ExoN domain

The ExoN activity of nsp14 is responsible for proofreading, an unusual feature in the realm of +RNA viruses (51). Due to the very large genome of coronaviruses (∼ 30 kb) relative to other +RNA viruses, a proofreading activity is necessary to avoid mutational catastrophe. Many RNA viruses including coronaviruses are sensitive to remdesivir, a delayed chain terminator that is incorporated in viral RNA (52–55). The nsp14 ExoN activity also removes nucleotide inhibitors incorporated into the viral RNA. This is supported by an observation that SARS-CoV mutants with an inactive ExoN domain accumulated mutations and were more sensitive to remdesivir and 5′-fluorouracil (56–59). A largely increased potency of remdesivir (in cell cultures) against SARS-CoV-2 was observed when combined with an ExoN inhibitor such as the hepatitis C virus NS5A inhibitors, pibrentasvir and ombitasvir, that were identified as potent SARS-CoV-2 ExoN inhibitors (60). Notably, nsp14 also forms a complex with the activating protein nsp10, which is essential for its ExoN activity but not for its MTase activity (61). A high resolution structure of the SARS-CoV-2 nsp14 ExoN domain in complex with nsp10 was reported recently (49). It revealed the location of the catalytic tetrad DEED (Asp90, Glu92, Glu191, Asp271) and the magnesium cation, and confirmed previous observation with the SARS-CoV nsp10/nsp14 structures (32,47) that the surface of nsp10 that interacts with nsp14 largely overlap with the nsp10 surface interacting with nsp16. Therefore, one nsp10 molecule cannot interact with nsp14 and nsp16 at the same time (49). However, nsp10 is not a limiting factor due to ORF1a's increased expression (62). Very recently a cryo-EM structure of the nsp10/nsp14 complex with RNA was reported and shed light on the RNA binding mode in the ExoN active site (50).

#### The N7-MTase domain

The N7-MTase domain of nsp14 is located at its C terminus and, interestingly, the N-terminal ExoN domain is important for its enzymatic activity (63). However, unlike the nsp16, the N7-MTase domain does not need nsp10 to be active and its fold is unusual. It is composed of 12 β-strands and 5 α-helices, its central β-sheet is composed of five β-strands (β11, 12, 14, 15, 22), and is surrounded on one side by an α7 helix and on the other by three long loops that bear two very small helices (α8 and η3), where this part of the MTase domain makes up most of the SAM binding site. The hinge region (N-terminal loop consisting of residues 288–300 and β-strands 16–18) and the C-terminal part (α9, 10, β 20–22) constitute the pre-capped RNA (GpppRNA) binding site that is also stabilized by a zinc finger (Figure 8) (47,50). The key residues for its enzymatic function were identified by alanine scanning (31), and most of them are involved in the binding of pre-capped RNA (GpppRNA), or are a part of the SAM binding site D(I/V)GNPK(A/G) (residues 331–336) that is conserved among coronaviruses (Figure 8C, Supplementary Figure S2).

#### Role of the nsp14 within the replication-transcription complex (RTC)

**Figure 9.**
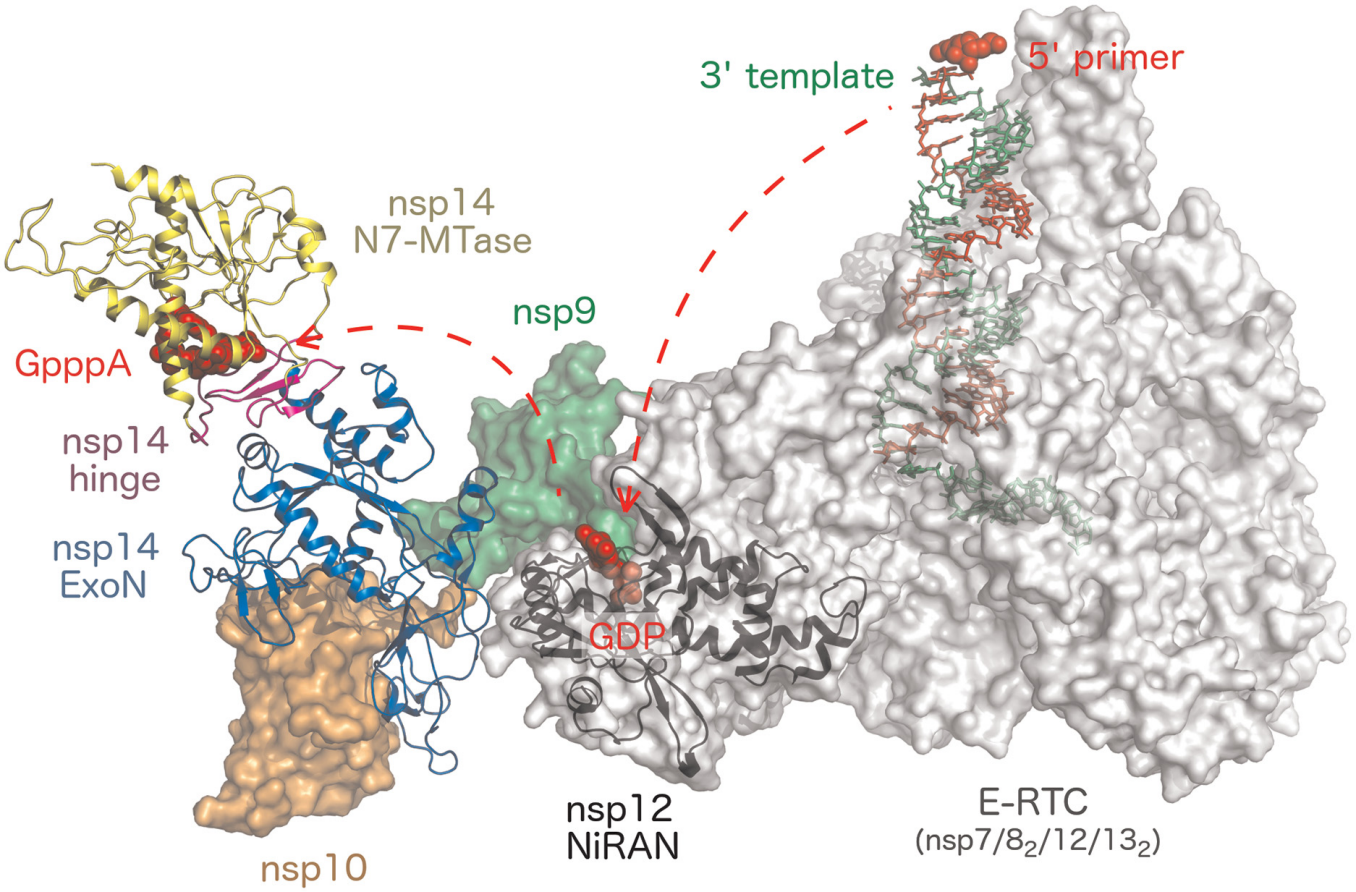
Assembly of the SARS-CoV-2 co-transcriptional capping complex. Elongation replication-transcription complex (E-RTC) composed of nsp7, nsp8, nsp12, and nsp13 is shown as a grey surface. The NiRAN subdomain of nsp12 is colored black in cartoon representation. The template and primer RNA strands bound to E-RTC are shown as green and red sticks, respectively. The single-stranded RNA binding protein nsp9 is shown as a green surface. The nsp10/nsp14 complex is depicted as in Figure 8, i.e. nsp10 is shown as an orange surface, while nsp14 is shown in cartoon. The figure was generated using the structures with PDB ID: 7EGQ (17), 7CYQ (12) and 5C8S (32).

The exact composition of the RTC is not yet clear, nevertheless, multiple models based on both cryo-EM experimental data and *in silico* docking studies are emerging. One postulated model based on *in silico* docking studies proposed by Perry et al. suggests that the RTC could be formed by a large protein assembly (18) around the hexameric endonuclease (nsp15), composed of the RdRp (RNA-dependent RNA polymerase, nsp12/nsp7/(nsp8)_2_), the capping and ExoN enzymes (nsp14/nsp16/(nsp10)_2_), the helicase (nsp13), nsp9 and the nucleocapsid (N) protein (12,17,18,64–71). It should be noted that this nsp15-centered hexameric model does not align with the structure based on cryo-EM experimental data reported by Yan *et al.* (17). This cryo-EM structure suggests a SARS-CoV-2 co-transcriptional capping complex composed of the RdRp, helicase, nsp9, and the nsp14/nsp10. It revealed the binding mode of the nsp14/nsp10 complex to the RdRp and the nsp9 protein (Figure 9). The structure suggests that the NiRAN domain of nsp12 is the most important for the formation of this complex. The interface is composed of a NiRAN domain, nsp9 and the ExoN domain of nsp14. The NiRAN domain is responsible for pre-capped (GpppRNA) synthesis, it catalyzes the GTP transfer. As the RNA polymerization continues, the pre-cap structure can reach the nsp14 MTase active site where the cap-0 would be synthesized (*cis* mechanism). However, given the rather large distance between the NiRAN and MTase active sites, it is quite possible that another co-transcriptional capping complex is involved in cap-0 synthesis (*trans* mechanism). The ExoN catalytic site is located ∼80 Å away from the polymerase active site and 120 Å away from the dsRNA exit site, implicating the *trans* mechanism of proofreading (17). However, it must be noted that this structure was obtained using a covalently linked nsp9 and nsp10 protein (nsp9-nsp10 fusion protein) and the nsp9 N terminus contained additional four amino acid residues as a result of a cloning artifact. Such a non-native N terminus of the nsp9 cannot undergo UMPylation by the NiRAN domain which would be lethal for the virus (72). It is tempting to speculate that the structures reported by Yan et al. represent an intermediate replication-elongation complex that exists before the UMPylation of nsp9.

### Inhibitors of the coronaviral MTases

Although the importance of these enzymes has been known for almost two decades (73,74), selective inhibitors are not yet known for MTases from SARS-CoV or any other coronaviruses. In most cases the compounds are either nonspecific inhibitors of coronaviral MTases or the data for their specificity are missing. In this review, we have tried to cover inhibitors for which the authors were able to determine their biological activity either on target proteins (nsp14 or nsp16) *in vitro* or in cell cultures. However, to our best knowledge, none of these compounds was tested in animals. Inhibitors that have been shown to inhibit either nsp14 or nsp16 MTases can be divided into four groups. The first group consist of derivatives which occupy the SAM binding site (SAM-competitive inhibitors). These compounds are usually derived from SAM or SAH by a slight modification of their chemical structure. The second group is composed of several derivatives of an RNA cap that can inhibit either nsp14 or nsp16. Next, a small group of compounds acts as inhibitors of protein-protein interaction between nsp16 and its crucial cofactor nsp10. Finally, there are a number of compounds that have been identified in high-throughput screens against either nsp14 or nsp16 where the mechanism of action is not entirely clear.

#### Inhibitors targeting the SAM binding site of coronaviral MTases

The use of SAM as a methylation agent is not specific to viruses; SAM is also used by many cellular enzymes. Therefore, there is a need to develop SAM analogues specific for viral MTases. Although achieving high selectivity is always difficult, the binding site for SAM has been already exploited for the design of novel human MTase inhibitors and it has resulted in several promising selective drug candidates (75). From a medicinal chemistry perspective, the SAM molecule can be divided into three parts: the amino acid residue, sugar moiety and the adenine nucleobase.

There are two naturally occurring compound that highly resemble a SAM molecule that have been used as a standard control for most of the MTase inhibitory assays: SAH and sinefungin. Both of these compounds can be regarded as amino acid residue modifications.

SAH is a by-product of SAM-dependent methylation. It differs from SAM only in the absence of a methyl group on the amino acid part, and it functions as a competitive inhibitor of MTases. Sinefungin was first isolated from the bacterium *Streptomyces griseolus* and presented as an antifungal agent (76). From a chemical perspective, sinefungin resembles SAM, although the positive charge intrinsically present on the sulfur atom of SAM is mimicked by an amino group of sinefungin that may be protonated under physiological conditions. Therefore, the mechanism of action of sinefungin is based on its ability to inhibit MTases, namely to prevent SAM binding to its binding site. Shortly after its discovery, it was shown that sinefungin is capable of inhibiting not only fungal MTases, but also viral and mammalian MTases (77,78). Thus, it was logical to use SAH and sinefungin in the first experiments with coronaviral MTases as well. Bouvet et al. were the first to show that both SARS-CoV MTases can be inhibited by SAH and sinefungin (15). SAH exerted significantly lower activity against both nsp14 (IC_50_ = 16 μM) and nsp10/nsp16 (IC_50_ = 12 μM) in comparison to sinefungin (nsp14 IC_50_ = 496 nM, nsp10/nsp16 IC_50_ = 736 nM). Aouadi *et al.* showed that these two compounds can also inhibit nsp10/nsp16 from MERS-CoV (79). Recently, SAH and sinefungin were shown to inhibit both SARS-CoV-2 MTases and both are widely used as baseline standards for assays related to these coronaviral proteins (80–83).

It can be assumed that derivatives with an altered amino acid moiety will play one of the major roles in the future development of new derivatives targeting the SAM binding site both at nsp14 and nsp10/nsp16. At present, however, the arsenal of such compounds is rather limited and only a few compounds targeting either nsp14 or nsp10/nsp16 form SARS-CoV-2 have been reported.

**Figure 10.**
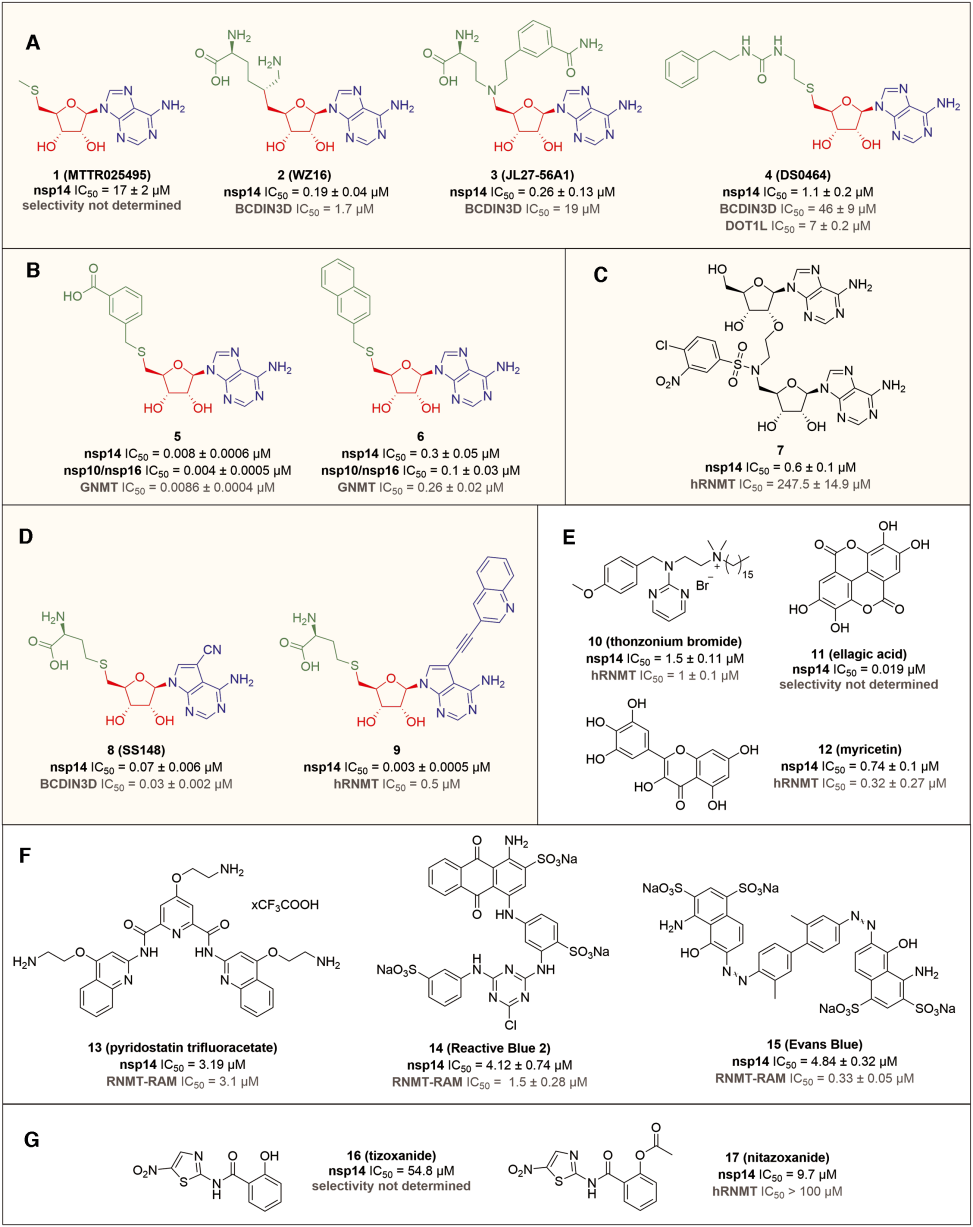
Inhibitors of coronaviral MTases and their IC_50_ values and selectivity based on enzymatic assays. (**A**) SAM analogues with modified amino acid moiety (1–4). (**B**) Non-specific SAM analogues with more lipophilic substituents **5** and **6**. (**C**) Adenine dinucleoside inhibitor **7**. (**D**) SAH analogues **8** and **9** with modified nucleobase. (**E**) The most active compounds (10–12) discovered by HTRF (homogeneous time resolved fluorescence) assay. (**F**) Three compounds (13–15) with antiviral effect on SARS-CoV-2. (**G**) Tizoxanide **29** and nitazoxanide **30**. Inhibitors are divided into colored boxes according to their structural motive: yellow – SAM derivatives, green – structure mimicking the transit state, purple – random structures received by HTS. Color-coding of SAM analogues structures is: green – amino acid moiety, red – sugar part and blue – nucleobase. BCDIN3D – bicoid interacting three domain containing RNA MTase, DOT1L – disruptor of telomeric silencing-1 like histone lysine MTase, GNMT – glycine N-MTase, hRNMT – human RNA N7-MTase, RNMT-RAM – complex of RNMT and RNMT-activating miniprotein.

Screening by Devkota et al. revealed several such derivatives as inhibitors of nsp14 (81). These results indicate that removal of the amino acid moiety leads to a remarkable loss of activity (see compound **1**, Figure 10A). However, other sinefungin derivatives exemplified by **2**, which differs by one methylene bridge compared to sinefungin, also show significant inhibitory activity against nsp14. Similarly, further modifications of this part of the molecule, such as substituting of the sulphur atom of SAH for nitrogen combined with the introduction of a lipophilic chain in this position as in the case of **3** or substitution of the amino acid part for the urea part as in the case of **4**, may lead to a significant inhibitory effect. They also examined selectivity of **4** against numerous human MTases and showed that the compound exert inhibition of only the histone DOT1L MTase with selectivity index lower than 10 (Figure 10A).

Bobileva et al. showed that a relatively simple substitution of the SAH amino acid part can result in highly active inhibitors of both SARS-CoV-2 MTases (84). They examined various potential bioisosteres of this moiety and proved that significantly more lipophilic derivatives such as compounds **5** and **6** exert a more profound effect against both enzymes. Unfortunately, they also showed that the compounds inhibit human GNMT MTase as well and no further selectivity studies were conducted (Figure 10B).

As a specific case of modification of this part of the molecule, the bisubstrate analogues prepared by Ahmed-Belkacem et al. can also be considered (85). They published 16 different adenine dinucleosides that mimicked the transit state of the 2′-*O* methylation of the RNA cap. They linked the nucleosides together via a nitrogen-containing linker. None of the 16 compounds was an effective inhibitor against the 2′-*O*-MTases of several viruses in the *Poxviridae* or *Flaviviridae* family or SARS-CoV. However, seven compounds inhibited the N7-MTase activity of SARS-CoV nsp14, six of them at micromolar concentrations and one (compound **7**) even at submicromolar concentrations. The authors were able to show that these inhibitors do not inhibit human N7-MTase (RNMT), however, selectivity towards other human MTases has not been studied (Figure 10C).

To our knowledge, no coronavirus MTases inhibitors containing a modified sugar moiety have been described so far. The original sugar, ribose, seems be the best fit for the SAM binding site, although at least one exception was described (86). Notably, various MTases show different sensitivity to a modification of this ribose moiety which could be utilized as a source for selectivity (87). The ribose moiety was also used as a site to attach a fluorescent tag to SAM producing an useful chemical-biology tool (88).

Finally, several inhibitors derived from SAH molecules were reported recently. Devkota et al. identified in their screening campaign mentioned above a 7-cyano-7-deaza derivative of SAH **8** (Figure 10D) that exerted double digit nanomolar activity against nsp14 from SARS-CoV-2 (81). At the same time, our team was engaged in the rational design of nsp14 inhibitors and concluded on the basis of the nsp14 SARS-CoV crystal structure that modified 7-deaza SAH derivatives bearing various hydrophobic substituents attached to position 7 by linkers can potentially inhibit this enzyme. Based on this design, we prepared a series of novel inhibitors, such as **9** (Figure 10D) that exhibited significantly higher inhibitory activity of nsp14 compared to sinefungin (80).

#### Inhibitors targeting the RNA binding site of coronaviral MTases

RNA is the actual substrate that is methylated by both coronaviral MTases. Therefore, blocking its binding to these proteins is one possible approach to designing new inhibitors. However, only a handful of substances derived from the RNA cap structure are currently known to be able to inhibit this binding (15,79).

Several studies have shown that RNA can be effectively displaced from nsp10/nsp16 complex by N7 methylated dinucleotide analogues of the cap (e.g. N7-methyl-GpppG), whereas non-methylated analogues were inactive (15,79). Although these analogues are in principle potent inhibitors of the nsp10/nsp16 *in vitro*, their application *in vivo* will be always complicated by their polyionic nature.

#### Inhibitors targeting nsp10/nsp16 protein-protein interaction

The nsp10-nsp16 interaction is complex and its total area in SARS-CoV-2 is 1983 Å^2^ (38) suggesting that disrupting this interaction with a small molecule would be quite difficult. However, the fact that the nsp10 and nsp16 proteins are highly conserved across β-coronaviruses, including Feline CoV, SARS-CoV, SARS-CoV-2 and other (Supplementary Figure S1), makes this interaction a predictor for the development of broad-spectrum antivirals.

Ke *et al.* were the first to show that the interaction between nsp10 and nsp16 from SARS-CoV can be disrupted by small peptides derived from nsp10 protein (89). They showed that two small peptides (K12 and K29 having 12 and 29 amino acids, respectively), are able to inhibit 2′-*O*-MTase activity in dose-dependent manner with IC_50_ approximately 160 μM.

Wang and colleagues confirmed this using a peptide (P29) that consisted of a segment of nsp10 (amino acids 68–96) from the murine hepatitis virus (MHV) (73). *In vitro* incubation of the nsp10-nsp16 complex from MHV, IBV (infectious bronchitis virus), SARS-CoV and MERS-CoV together with P29 resulted in a significant inhibition of MTase activity by >50%. For P29 to be effective in cells, it was necessary to fuse P29 with the Tat protein from HIV (TP29), as the Tat allowed the peptide to cross the cytoplasmic membrane. The cell culture was infected with MHV strain A59 (MHV-A59) and TP29 was added one hour after infection. Analysis after 20 hours showed an 80% lower viral titer compared to the control. The authors think that the peptide inhibits the formation of the nsp10/nsp16 protein complex, leading to inhibition of genome replication and deficiency of caps on RNA. Consequently, without the cap, proper translation does not occur, and the cytosolic receptors of cap-0 mRNA trigger a cellular response in the form of interferon production. The advantage of using TP29 is that it targets a structure that is specific to coronavirus nsp16 and therefore the treatment should result in lower incidence of side effects.

#### Inhibitors of coronaviral MTases identified by high-throughput screening

Several high-throughput screening (HTS) campaigns against both nsp10/nsp16 and nsp14 leading to a number of structurally diverse hit compounds has been reported recently.

To our knowledge, the first complex screening for SARS nsp14 and nsp16-nsp10 inhibitors was performed by Aouadi et al. that tested two thousand compounds composed of FDA approved drugs, natural products and various pyridazine derivatives (90). They used a Homogeneous Time-Resolved Fluorescence (HTRF) assay for the initial nsp14 screen of the compounds and a radioactive-based evaluation of the obtained hit compounds. Their study identified twenty hit compounds that were further evaluated by radioactive MTase assays for inhibition of nsp14 from MERS and nsp10/nsp16 from SARS and MERS as well as several flaviviral MTases and human RNMT. The most profound effect was exerted by compounds **10**, **11** and **12** (Figure 10E).

Recently, a team of Prof. Jemielity has published a very interesting and complex study on novel nsp14 inhibitors identified by HTS fluorescence-based assay (91). They have not only performed the screening of more than seven thousand compounds from various libraries, but have also evaluated the hits from enzyme assay in cell-based antiviral assay. Their study shows that although a numerous types of chromone, antraquinone, 2-aminothiophene-3-carbaldehyde, naphthalene, and biphenyl derivatives can potently inhibit the isolated enzyme *in vitro*, their activity in cells is rather limited. Of the 83 identified inhibitors that had an IC_50_ less than 50 μM, only three compounds **13**–**15** showed an antiviral effect. This team was also able to prepare an interesting assay to recognize the binding site of these inhibitors based on fluorescent probes. The structures of the compounds active in cells are summarized in Figure 10F.

Finally, Pearson et al. introduced a very elegant mass spectrometry-based methodology for screening nsp14 inhibitors (92). In this assay, they screened more than 1700 compounds and identified tazoxanide **16** and nitazoxanide **17** as other potential hit compounds for further optimization (Figure 10G). The activity of nitazoxanide was confirmed by orthogonal radioactive assay (with an approx. 10-fold shift in activity). This substance had been previously identified as a coronavirus replication inhibitor, but the mode of action of this derivative seems to be complex.

Several other HTS assays for both nsp14 and nsp10/nsp16Pfizer

were utilized to identify SAM/SAH and RNA derived inhibitors described above. These methodologies can serve as important tools for identification of novel compounds with a high potential to serve as starting points for serious research for SARS-CoV-2 and other coronavirus therapeutics.

## CONCLUSION

Vaccination is the main weapon to combat COVID-19. Nonetheless, small molecule-based drugs are urgently needed for people who, for various reasons cannot get vaccinated or who acquire COVID despite being vaccinated. Many new assays to discover small molecules to inhibit coronaviral enzymes and many new small molecules inhibiting coronaviral enzymes, including both proteases, RdRp, the endonuclease (nsp15), ExoN activity and the helicase (nsp13) were reported recently (60,80–82,90–99). PF-07321332, an orally available protease inhibitor by Pfizer, was very recently FDA-approved for emergency use. Recently an orally available prodrug of remdesivir was reported (100–102). Two other orally available nucleoside analogs: molnupiravir (a prodrug of N4-hydroxycytidine) (103) and AT-527 (a prodrug of a guanosine nucleotide analog) (104) were described to be effective against SARS-CoV-2 and molnupiravir is currently approved in several countries. Both coronaviral MTases are vital for the virus to evade innate immunity, and their inhibitors are developed by us and others. However, there are still several challenges that remain to be resolved for successful targeting of both coronaviral MTases with appropriate therapeutics. Firstly, it involves solving the structures of both nsp10/nsp16 and nsp14 from SARS-CoV-2 and other coronaviruses with selective small molecule inhibitors targeting both SAM and RNA binding sites to gain structural information on their mechanism of action. And, secondly, to use that information and optimize these inhibitors to be able to efficiently cross cell membranes and to selectively target viral MTases. To successfully treat a viral infection, multiple drugs are often needed at the same time so that the virus cannot develop escape mutations. Hopefully, further research into MTase inhibitors will lead to future successful treatments against COVID-19 and other potentially deadly viral infections.

## Supplementary Material

gkab1279_Supplemental_File
