# Supplementary material for: Coronaviral RNA-methyltransferases: function, structure and inhibition
Source: Nucleic Acids Res. 2022 Jan 8;50(2):635–50. doi: 10.1093/nar/gkab1279 (PMC8789044; doi:10.1093/nar/gkab1279)
Supplement: gkab1279_Supplemental_File [file gkab1279_supplemental_file.pdf]

Coronaviral RNA-methyltransferases: Function, structure, and inhibition

Radim Nencka[a,*], Jan Silhan[a], Martin Klima[a], Tomas Otava [a], Hugo Kocek [a], Petra Krafcikova[a], Evzen Boura[a,*]

[a]Institute of Organic Chemistry and Biochemistry, Academy of Sciences of the Czech Republic, v.v.i, Flemingovo nám. 2, 166 10 Prague 6, Czech Republic.

[*] correspondence to nencka@uochb.cas.cz or boura@uochb.cas.cz

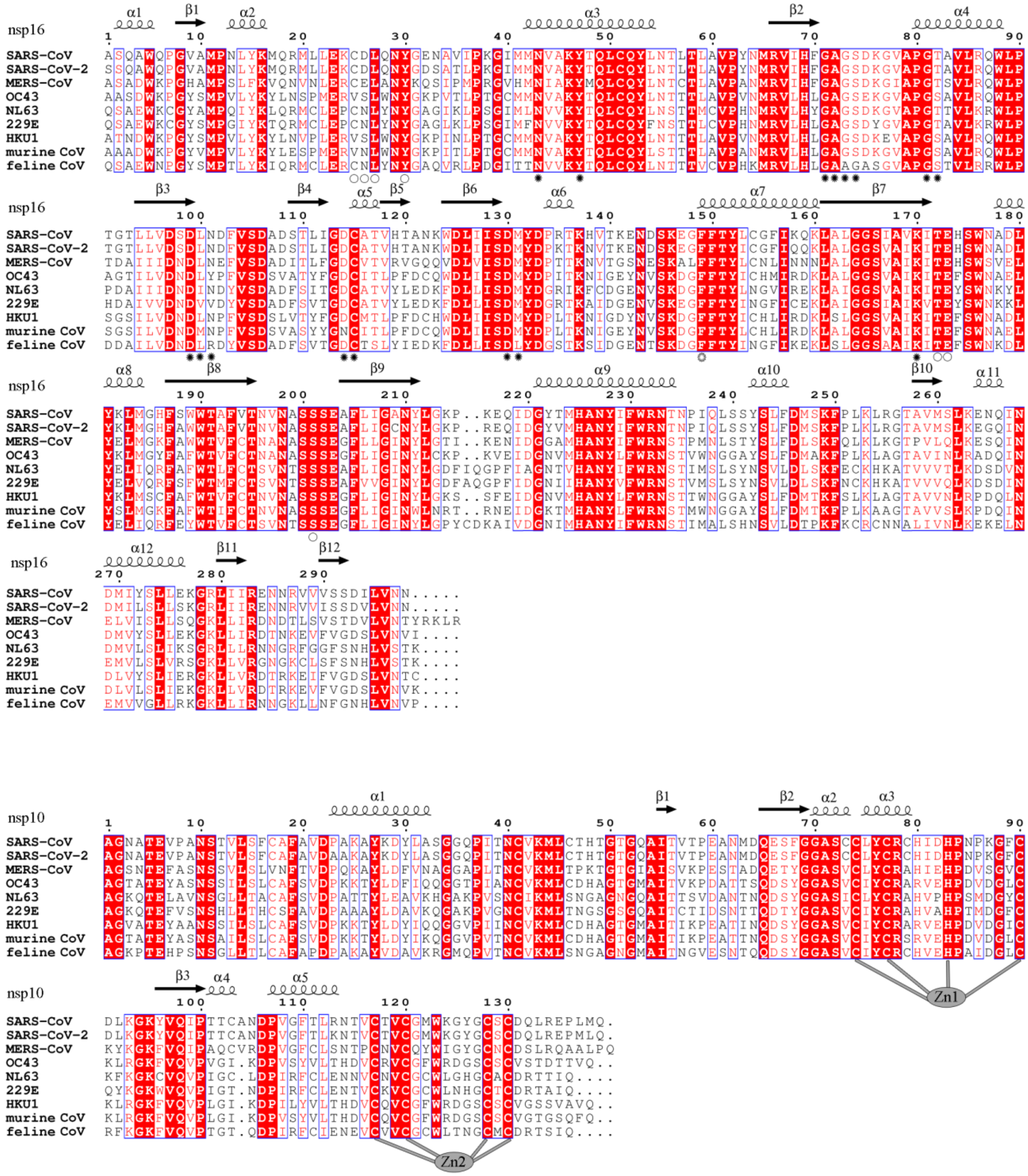

SI Figure 1. **Primary sequence alignment of selected coronaviral 2′-O-MTases (nsp10/nsp16 protein complexes)**. Nsp16 in the upper panel, nsp10 in the lower panel.

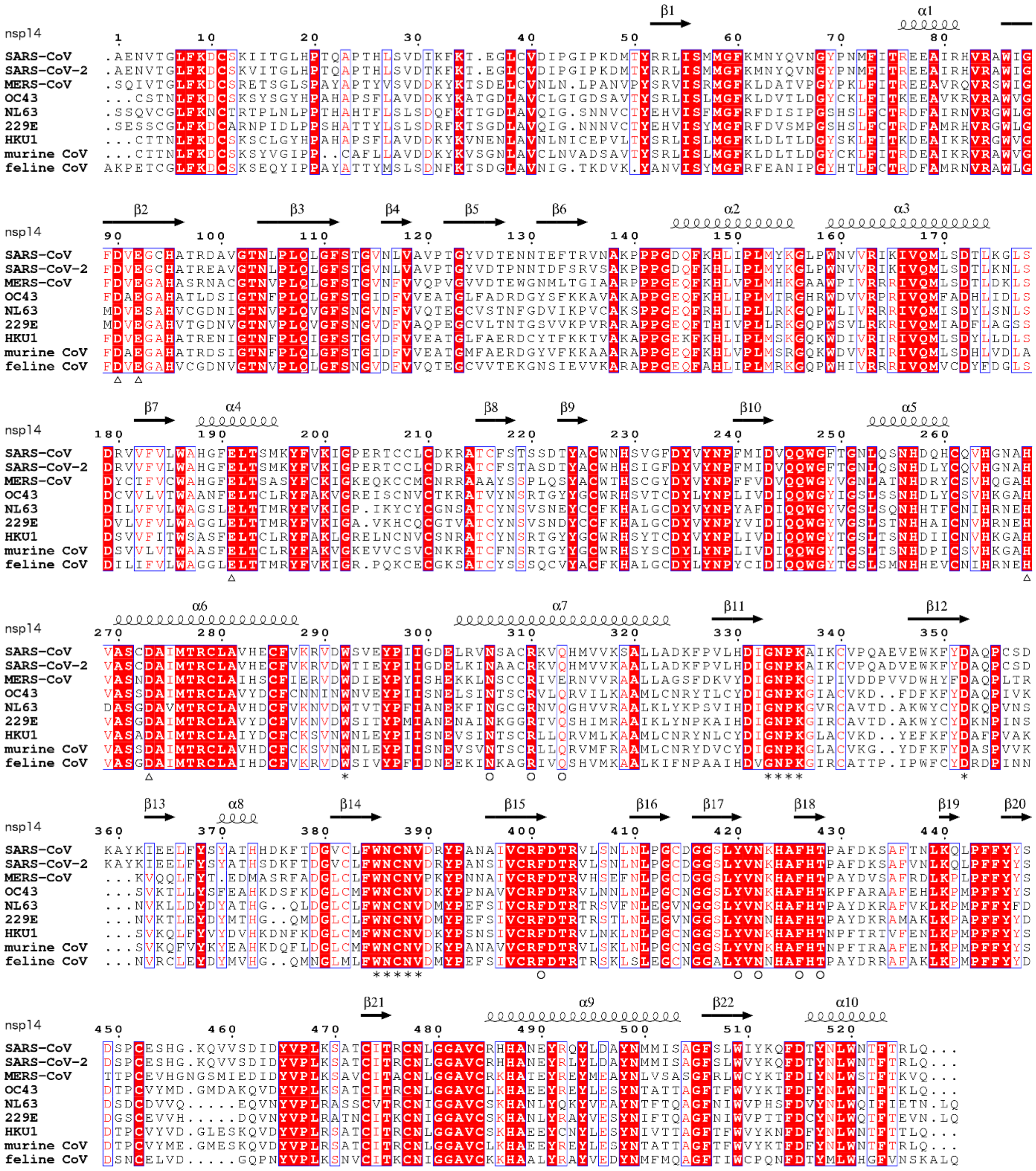

SI Figure 2. **Primary sequence alignment of selected coronaviral N7-MTases (nsp14 proteins).**

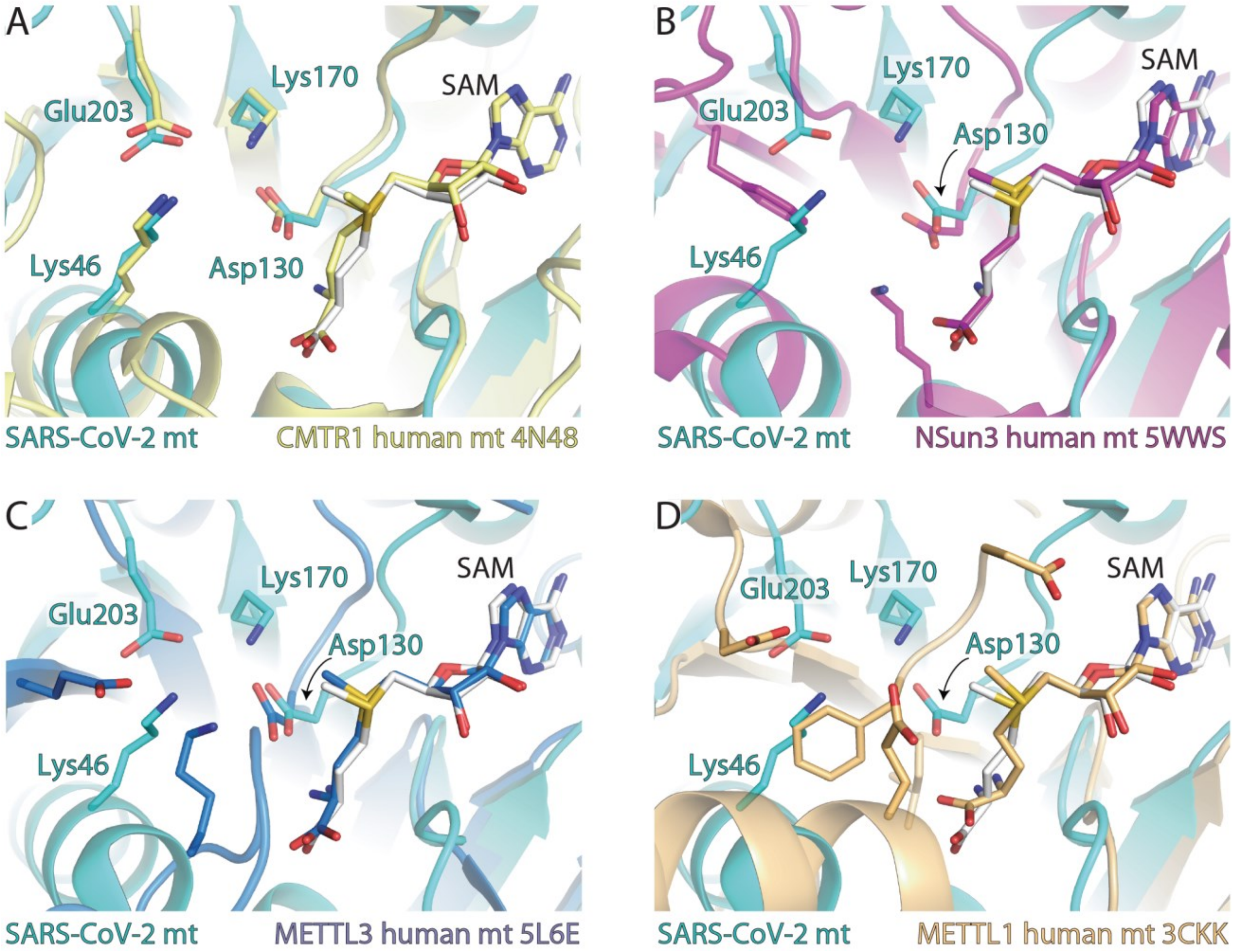

SI Figure 3. **Structural comparison of SARS-CoV-2 nsp16 catalytic tetrad with selected human MTases** Conserved residues of the catalytic tetrad of SARS-CoV-2 and those in the vicinity of S-adenosylmethionine (SAM) are shown in sticks. A) Detail of catalytic tetrad of 2′-O-MTases from coronavirus and human CMTR1 (Cap-specific mRNA (nucleoside-2'-O-)-methyltransferase 1, PDBID = 4N48 in pale yellow), B) an overlay with human RNA:m5C methyltransferase, NSUN3 (NOP2/Sun RNA Methyltransferase 3, PDBID = 5WWS), only one residue close to Asp130 resembles a part of the catalytic tetrad. C) Comparison with human METTL3 (N6-adenosine-methyltransferase 3,70 kDa subunit of methyltransferase, PDBID = 5L6E in light blue). D) Comparison with human tRNA guanine-N(7)-)-methyltransferase METTL1 (methyltransferase-like protein 1, PDBID = 3CKK light orange).

SI Materials & Methods

Sequences were obtained from UniProt as follows: SARS-CoV (UniProt entry P0C6X7), SARS-CoV-2 (P0DTD1), MERS-CoV (K9N7C7), OC43 (P0C6X6), NL63 (P0C6X5), 229E (P0C6X1), HKU1 (P0C6X2), murine CoV (strain MHV-JHM; P0C6Y0), feline CoV (strain FIPV WSU-79/1146; Q98VG9). Sequence alignment was performed with ClustalX 2.0 [1] and rendered with ESPript 3.0 [2]. Red areas represent the identical residues, while the conserved residues are highlighted by the red font. Secondary structure elements are indicated above the sequences.

1. Larkin, M.A., et al., *Clustal W and Clustal X version 2.0.* Bioinformatics, 2007. **23**(21): p. 2947-8.
2. Robert, X. and P. Gouet, *Deciphering key features in protein structures with the new ENDscript server.* Nucleic Acids Res, 2014. **42**(Web Server issue): p. W320-4.
